# Costs of staged versus simultaneous bilateral total knee arthroplasty: a population-based study of the Taiwanese National Health Insurance Database

**DOI:** 10.1186/s13018-014-0059-6

**Published:** 2014-07-15

**Authors:** Aaron Chih-Chang Lin, En Chao, Che-Ming Yang, Hsyien-Chia Wen, Hsiao-Li Ma, Tzu-Chuan Lu

**Affiliations:** 1 Department of Orthopedics, Songshan Branch, Tri-Service General Hospital, National Defense Medical Center, Taipei 105, Taiwan; 2 Institute of Clinical Medicine, National Yang-Ming University, Taipei 112, Taiwan; 3 National Defense Medical Center, Taipei 114, Taiwan; 4 School of Health Care Administration, Taipei Medical University, Taipei 110, Taiwan; 5 Department of Orthopedics, Taipei Veterans General Hospital, Taipei 112, Taiwan; 6 Taipei Medical University Shuang Ho Hospital, New Taipei 235, Taiwan

**Keywords:** Total knee arthroplasty, National Health Insurance Research Database, Staged bilateral TKA, Simultaneous bilateral TKA

## Abstract

**Background:**

Bilateral total knee arthroplasty (TKA) is required for many patients. There are few studies comparing the overall costs of staged and simultaneous bilateral TKA.

**Methods:**

The Taiwan National Health Insurance Research Database (NHIRD) was searched, and the total medical costs of 452 patients who received simultaneous bilateral TKAs were compared with those of 690 who received staged bilateral TKAs.

**Results:**

All categories of medical costs were lower in the simultaneous TKA group, with the exception of therapeutic procedure fees which were higher in the simultaneous bilateral TKA group. The 10-year prosthesis survival rates for simultaneous and staged bilateral TKA were 90.9% and 87.5% (*p* > 0.05), respectively.

**Conclusions:**

These results indicate that simultaneous bilateral TKA is more cost effective than staged bilateral TKA. Prosthesis survival is not affected by the choice for staged or simultaneous bilateral TKA.

## Background

Total knee arthroplasty (TKA) is widely believed to be the best choice for the treatment of end-stage of knee arthropathy, and the procedure can significantly improve the quality of life for these patients. Multiple diseases such as osteoarthritis (OA), rheumatoid arthritis (RA), and hemophilia can result in severe bilateral knee destruction, and patients frequently require bilateral TKA [1]. However, there is still debate regarding performing staged bilateral or simultaneous bilateral TKA, primarily because of the concern of increased complication rates with simultaneous bilateral TKA [2]-[11]. A recent meta-analysis by Restrepo et al. [4] reported that compared with staged bilateral and unilateral TKAs, simultaneous bilateral TKA was associated with higher risk of serious cardiac complications, pulmonary complications, and mortality. A meta-analysis by Hu et al. in 2011 [2], however, reported that although mortality and neurological complications were greater in patients who underwent simultaneous bilateral TKAs as compared with those who underwent staged bilateral TKAs, there was no difference between the two groups with respect to infection, pulmonary embolism, deep vein thrombosis (DVT), and cardiac complications.

A few studies have compared the relative costs of performing simultaneous versus staged bilateral TKAs; however, those that have generally report that simultaneous bilateral TKA is associated with lower costs than staged bilateral TKA [10]-[13]. While not a direct measure, medical costs, both inpatient and outpatient, can reflect the incidence of complications [4],[12],[14].

The National Health Insurance (NHI) was established in Taiwan in 1995. The National Health Insurance Research Database (NHIRD) provided by the Bureau of National Health Insurance, Department of Health, Taiwan, and managed by the National Health Research Institutes maintains data of all NHI medical benefit claims for the Taiwanese population of over 23 million, which represented over 99% of the island’s population in 2010. To the best of our knowledge, there is no other database like the Taiwan National Health Insurance NHIRD that complied such long-term complete population data [15].

The purpose of this study was to compare the medical utilization during hospitalization and 1 year after discharge and long-term prosthesis survival in patients who received simultaneous bilateral TKAs and staged bilateral TKAs using data in the Taiwan NHIRD.

## Methods

This research used pooled data for the years 1996 to 2010 obtained from NHIRD - Longitudinal Health Insurance Database (LHID). The LHID contains all the original claim data of one million beneficiaries that were drawn for random sampling from the NHIRD. There is no significant difference in the gender (χ^2^ = 0.008, *df* = 1, *p* = 0.05) distribution between the patients in the LHID and the original NHIRD [15]. The NHIRD includes registries of all hospitals and all board-certified physicians and details of inpatient and outpatient medical utilization for each patient in Taiwan. Thus, all hospitals in Taiwan were represented in this study. The NHIRD provides operation procedure codes and diagnosis codes for each patient, using the International Classification of Disease, Ninth Revision, Clinical Modification (ICD-9-CM). The NHIRD is without patient or physician identifiers, by authorization from the National Health Research Institutes (NHRI), Taipei, Taiwan. This study was approved by the Institutional Review Board of our hospital and by the Bureau of National Health Insurance, and the requirement of patient informed consent was waived.

The study sample was identified from the LHID and included all patients who received primary TKA procedures (ICD-9-CM procedure code 81.54) during the period from 1996 to 2010. Briefly, based on NHI case payment guidelines [15] for TKA, cases in which the prosthesis cost was lower than the payment were excluded first [16]. This was done because a lower price most likely indicates an error during data input. Data indicating malignant diseases, fractures, or with procedure codes of other major operations were also excluded. The percentages of cases excluded due to data input errors (cost of prosthesis lower than reimbursement cost, 58 records) and other major conditions treated simultaneously (41 records, including 24 tumors, 12 concomitant major operations, 2 cases of leukemia, and 3 fractures) were 0.77% (58/7,490) and 0.55% (41/7,490), respectively. As these percentages are low, the impact of the exclusion of these cases on the data analysis and results is likely minimal.

**Figure 1:**
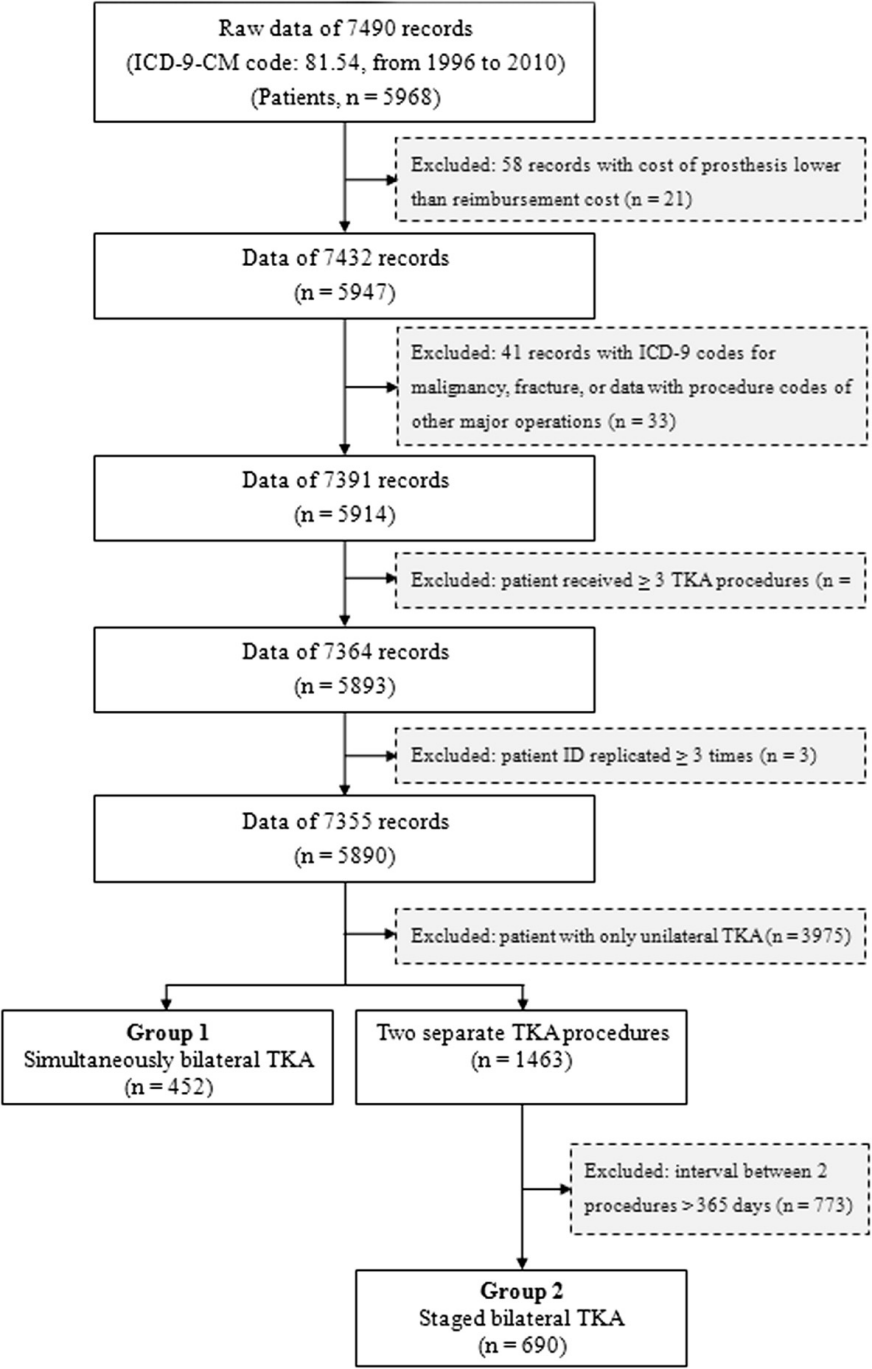
The flowchart of data collection.

The remaining data were then divided into two groups. Patients who underwent two TKA procedures separated by ≤365 days were considered to have undergone staged bilateral TKAs [7]. Cases in which the same patient identification number was identified in different hospitalization and the single surgical code 81.54 was associated with the hospitalizations were classified as staged bilateral TKA. Cases in which the procedure code 81.54 was identified twice during the same hospitalization were classified as simultaneous bilateral TKA. Using the above criteria, 452 cases were identified that received simultaneous bilateral TKAs and 690 that received staged bilateral TKAs. A flow diagram of patient selection is shown in Figure 1.

The medical expenditures of each claim were extracted and recorded. To reflect the real dollar value, all dollar values at the end of each year were adjusted to 2010 Taiwan currency values first, and then all dollar values were converted to US Dollars (USD) with the exchange rate of 1 USD = 31.64 New Taiwan Dollars (the averaged exchange rate from 1996 to 2010). To compare the medical utilization between the two groups, the costs in the simultaneous group were compared with the total costs of the two procedures in the staged group.

Pre- and postoperative medical utilization was determined from ambulatory care expenditures using the orthopedic outpatient department medical use costs. We calculated each claim’s average monthly orthopedic outpatient department medical use cost for 12 months before the operation to use as a reference value and determined when the monthly postoperative outpatient costs dropped below this value.

A failure of prosthesis was defined as a revision TKA (ICD 81.55) performed after a simultaneous bilateral TKA or after the second procedure of a staged bilateral TKA [8].

### Data analysis

Continuous variables were presented as medians and inter-quartile ranges (IQR, the range between the 25th and 75th percentile) due to non-normal distribution and were compared between the two groups (simultaneous bilateral TKA vs. staged bilateral TKA) by the Mann-Whitney *U* test. Categorical variables were expressed by counts and percentages and compared between different groups by the Chi-square test or the Fisher’s exact test, as appropriate. A linear mixed model was used to investigate the effects of TKA groups (group) and postoperative time (time) as well as their interaction (group × time) on medical costs during orthopedic outpatient visits after TKAs. A significant interaction (group × time) indicates the time trend of the medical expenditures during orthopedic outpatient visits differs between the two groups.

Kaplan-Meier survival curves were created to describe the prosthesis survival rates of the two groups. The time interval (month) between primary TKA and revision TKA was calculated in each group using a Kaplan-Meier survival curve, with 95% confidence interval (CI). The event time was calculated from the date of TKA completion to the date of the first revision during the follow-up. If no revision TKA was performed, the data were censored and the follow-up time was calculated until 31 December 2010. The log-rank test was used to compare the prosthesis survival rates between two TKA groups. Statistical analyses were performed with SAS software version 9.2 (SAS Institute Inc., Cary, NC, USA). A two-tailed *p* < 0.05 indicated statistical significance.

## Results

### Baseline characteristics

A total of 1,142 patients who received bilateral TKAs were included in the data analysis (Figure 1), consisting of 452 who received simultaneous bilateral TKAs (group 1) and 690 who received staged bilateral TKAs (group 2).

The demographic characteristics of the two groups are shown in Table 1. The patients who received simultaneous bilateral TKAs were older than those who received staged bilateral TKA at the first procedure (70.0 [65.0, 75.0] years vs. 69.0 [62.0, 73.0] years; *p* < 0.001). The gender distribution was neither different between the groups (group 1, 21.7% male vs. group 2, 23.0% male; *p* = 0.641) nor was the Charlson comorbidity index different between the groups (*p* = 0.848). The entire length of hospital stay was longer in the staged bilateral TKA group as compared to that of the simultaneous bilateral TKA group (15.0 [12.0, 18.0] days vs. 12.0 [9.0, 15.0] days, respectively; *p* < 0.001). Simultaneous bilateral TKAs tended to be conducted at a medical center (as opposed to regional or district hospitals) as compared to staged bilateral TKAs (46.5% vs. 37.7%, respectively; *p* = 0.012).

**Table 1:** Demographic and clinical characteristics of patients

	**Simultaneous bilateral TKA**	**Staged bilateral TKA**^**c**^	***p*** **value**
	**(*n*** **= 452)**	**(*n*** **= 690)**	
Age, years^a^	70.0 (65.0, 75.0)	69.0 (62.0, 73.0)	<0.001*
Gender, male^b^	98 (21.7)	159 (23.0)	0.641
CCI^b^			0.848
0	357 (79.0)	549 (79.6)	
1	85 (18.8)	123 (17.8)	
>1	10 (2.2)	18 (2.6)	
LOS, days^a^	12.0 (9.0, 15.0)	15.0 (12.0, 18.0)	<0.001*
Hospital type^b^			0.012*
Medical center	210 (46.5)	260 (37.7)	
Regional hospital	109 (24.1)	187 (27.1)	
District hospital	133 (29.4)	243 (35.2)	

CCI, Charlson comorbidity index; LOS, length of stay. ^a^Continuous data were presented as median (IQR) and compared by Mann-Whitney *U* test; ^b^categorical variables were expressed by counts and percentages and compared by the Chi-square test or the Fisher’s exact test, as appropriate; ^c^data was that from the first TKA except LOS, which was the summation of two admissions; asterisk indicated a significant difference between groups, *p* < 0.05.

### Medical utilization during hospitalization

**Figure 2:**
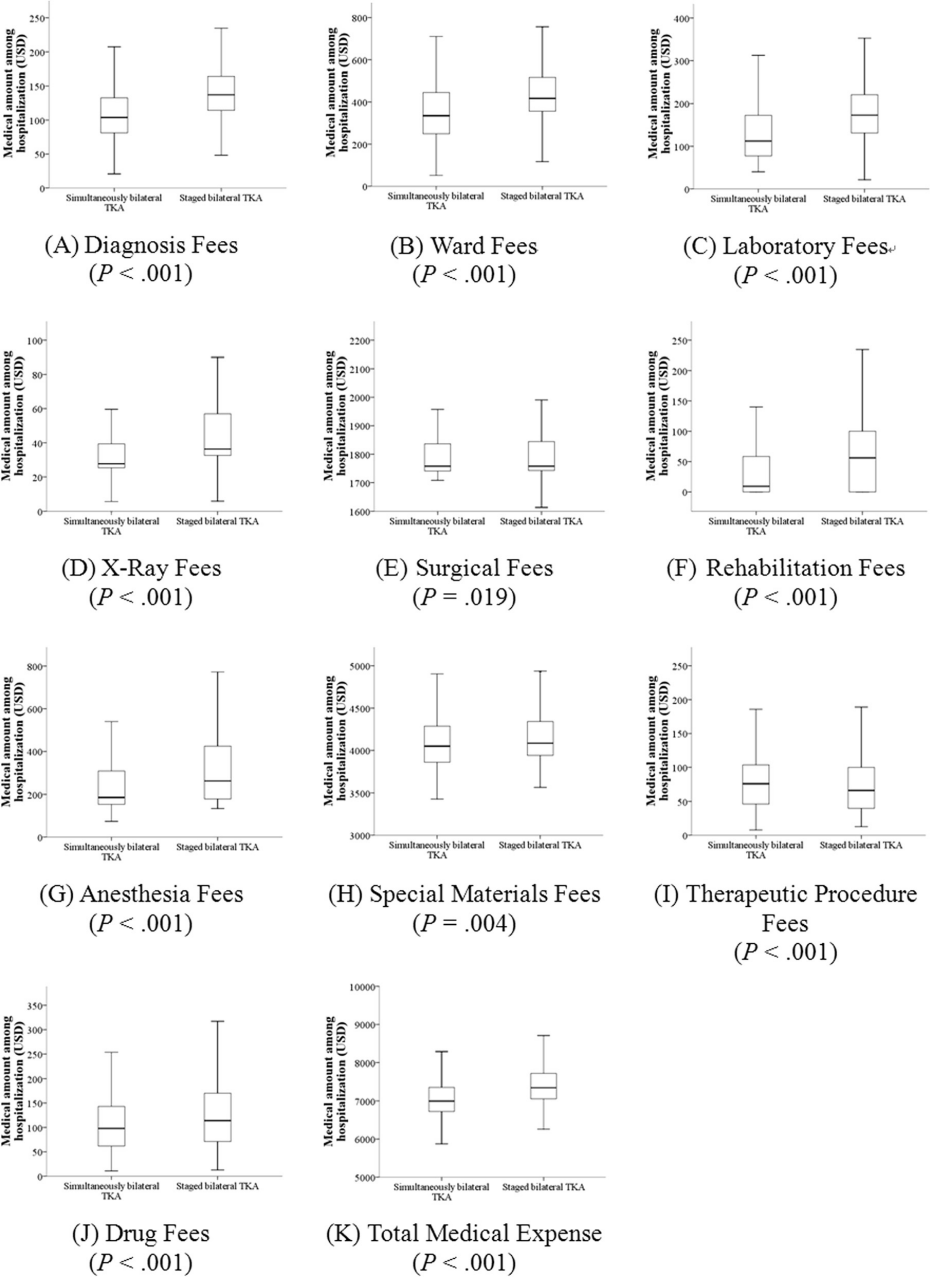
**Comparison of medical expenses during hospitalization between simultaneous and staged bilateral TKA.** According to different payment items, medical expenses during hospitalization between the two TKA groups (sum of the twice operations) were compared. **(A)** Diagnosis fees. **(B)** Ward fees. **(C)** Laboratory fees. **(D)** X-ray fees. **(E)** Surgical fees. **(F)** Rehabilitation fees. **(G)** Anesthesia fees. **(H)** Special materials fees. **(I)** Therapeutic procedure fees. **(J)** Drug fees. **(K)** Total medical expense. All *p* < 0.05.

The medical costs for each of the payment items during hospitalization are shown in Figure 2. For the staged bilateral TKA group, the sum of medical costs of the two stages was used in this analysis. The medical costs for almost all payment items during hospitalization were higher in the staged bilateral TKA group than in the simultaneous bilateral TKA group (all, *p* < 0.05). Therapeutic procedure fees, which include the medical costs for urethral catheterization, nasogastric tube placement, intravenous infusions, and dressing changes, however, were greater in the simultaneous bilateral TKA group (Figure 2I). It must be noted that in our country, therapeutic fees exclude the cost of the surgical procedure [17]. As a whole, the total medical cost during hospitalization was significantly higher in patients who received a staged bilateral TKA than in those who received a simultaneous bilateral TKA (7,345.7 USD [7,052.2, 7,721.7] vs. 6,994.4 USD [6,722.9, 7,354.1], respectively; *p* < 0.001, Figure 2K). Among all items, the highest costs were special materials fees (group 1 vs. group 2; 4,049.7 USD [3,859.8, 4,286.6] vs. 4,085.5 USD [3,940.8, 4,3411.7], respectively; *p* = 0.004, Figure 2H), and the lowest costs were X-ray fees (group 1 vs. group 2; 27.7 [25.3, 39.3] USD vs. 36.3 [32.6, 57.0] USD, respectively; *p* < 0.001, Figure 2D). Moreover, the total medical cost without special materials fees was also significantly higher in patients who received a staged bilateral TKA than in those who received a simultaneous bilateral TKA (3,245.5 USD [2,994.6, 3,509.9] vs. 2,947.9 USD [2,698.7, 3,215.1], respectively; *p* < 0.001).

### In-hospital mortality and complications

Two patients who received a simultaneous bilateral TKA expired during the postoperative period, while all patients who received staged bilateral TKAs survived to discharge. No significant difference of in-hospital mortality was found between the two groups (0.44% vs. 0.0%; *p* = 0.156). Data of specific complications (e.g., cardiac and pulmonary complications) are not available from the LHID/NHIRD data sets. However, the unplanned readmission rate may serve as an index of serious complications. To this end, we compared the unplanned readmission rates between the two groups at day 14, day 30, and day 90 after discharge (Table 2). Evaluation at 14 days was performed based on NHIRD guidelines which indicate that a second hospitalization within 14 days after surgery may be directly associated with complications of the index surgery. Evaluation at 30 and 90 days after surgery was based on prior studies [18],[19]. The analysis showed that the unplanned readmission rate at day 14 was significantly higher in simultaneously bilateral TKA than at day 14 after the first stage of staged bilateral TKA (2.88% vs. 0.87%; *p* = 0.016). Although the unplanned readmission rates at day 30 and day 90 were slightly higher in simultaneously bilateral TKA compared to that in staged bilateral TKA, no significant difference was found between two groups (all, *p* > 0.05).

**Table 2:** Unplanned readmissions in the two groups

**Postoperative day**	**Simultaneously bilateral TKA (*n*** **= 452)**	**Staged bilateral TKA (*n*** **= 690)**	
		**Stage I**	**Stage II**
14	13 (2.88)	6 (0.87)*	10 (1.45)
30	20 (4.42)	16 (2.32)	19 (2.75)
90	39 (8.63)	43 (6.23)	43 (6.23)

*p < 0.05 compared to simultaneously bilateral TKA as determined by Fisher’s exact test. Data are reported as number (percentage).

### Medical utilization during the first postoperative year

**Figure 3:**
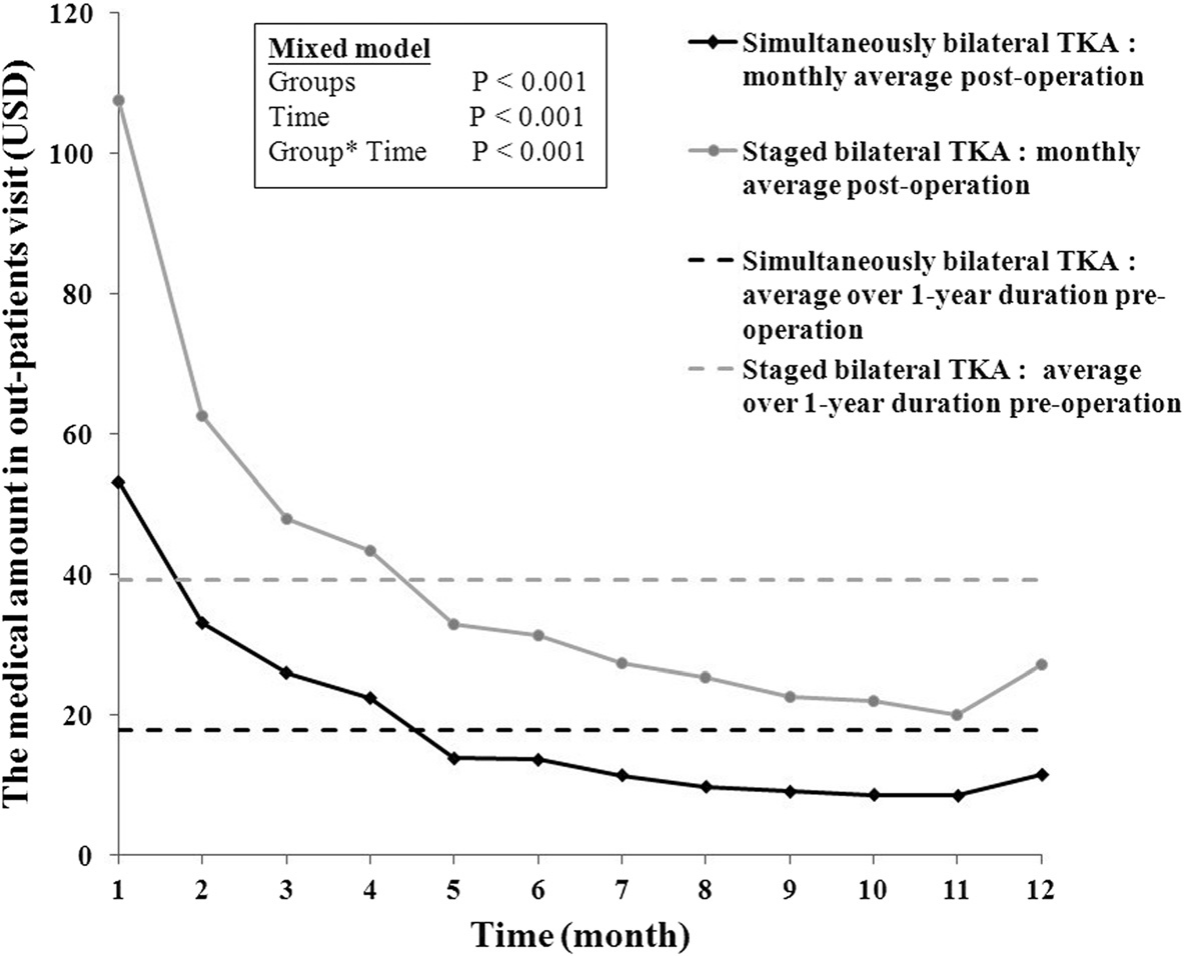
**Comparison of medical expenses during orthopedic outpatient visits over the first year postoperative period.** Medical expenses during orthopedic outpatient visits over the first year postoperative period between the two TKA groups were compared. The horizontal reference lines indicated the averages of medical amounts over 1-year duration pre-operation.

The trends of monthly average medical expenses incurred during orthopedic outpatient visits across the 1-year postoperative period are shown in Figure 3. In general, higher costs were incurred in the staged bilateral TKA group compared to the simultaneous bilateral TKA group (denoted as group effect; *p* < 0.001). Both cost trends decreased over time (denoted as time effect; *p* < 0.001), but a significantly greater rate of decrease in costs was seen in the group that received staged bilateral TKAs compared to those that received simultaneous bilateral TKAs (average decrease per month, 5.52 USD vs. 3.09 USD, respectively; *p* < 0.001). Five months after TKA, the average monthly medical costs were lower than the average of the 1 year prior to surgery for both groups.

### Prosthesis survival rate

**Figure 4:**
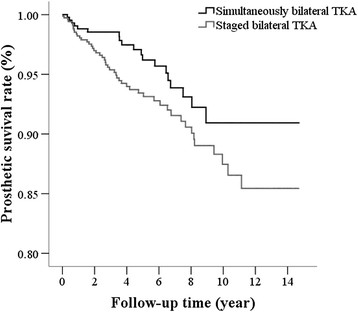
**Kaplan-Meier curve of the prosthesis survival of the simultaneous and staged bilateral TKA groups.** Log-rank test, *p* = 0.062.

There was no significant difference in the prosthesis survival rate between the two groups (log-rank test; *p* = 0.062, Figure 4). The 10-year prosthesis survival rates in the simultaneous bilateral TKA and the staged bilateral TKA groups were 90.9% (95% CI, 85.2% to 94.5%) and 87.5% (95% CI, 82.9% to 90.9%), respectively.

## Discussion

The NHIRD includes all patient medical benefit claims for NHI for the Taiwanese population of over 23 million, representing over 99% of the island’s population in 2010. The NHIRD has been used for a number of other studies in the field of orthopedics [14],[16],[20],[21]. The results of this study showed that medical costs are lower for simultaneous bilateral TKA as compared to staged bilateral TKAs. Even considering that the complication rate may be higher with simultaneous bilateral TKA, the costs are still lower than that of staged bilateral TKA.

The medical utilization costs for simultaneous bilateral TKA were significantly lower than for staged bilateral TKA, and this was primarily reflected in the total medical cost and average length of hospital stay. Our data showed that the entire length of hospital stay was longer in staged bilateral TKA group than in the simultaneous bilateral TKA group (15.0 days vs. 12.0 days), and the total medical expenses during hospitalization were significantly higher in the staged bilateral TKA group than in the simultaneously bilateral TKA (7,345.7 USD vs. 6,994.4 USD). Most, but not all of the special material fees, were due to the cost of the implant. Due to limitations of the database, we were not able to identify the individual implant cost; however, when the special material fee was deducted from the total medical cost, the cost was still significantly higher for staged bilateral TKA then simultaneous bilateral TKA.

In an early study, Reuben et al. [12] reported that the total costs of bilateral simultaneous TKA were significantly less than that of performing staged bilateral TKA and that the savings can exceed 10,000 USD for each patient. Stubbs et al. [10] performed a retrospective study at a community hospital and reported no difference in surgical or medical complications between simultaneous and staged bilateral TKAs and that overall costs were lower in the simultaneous TKA group. Kovacik et al. [22] also reported no difference in complications and a lower cost with simultaneous bilateral TKA, and March et al. [13] reported that although patients who received simultaneous TKAs had more postoperative complications, primarily thrombotic, they reported better physical function and general health in the first year postoperatively, and the overall costs were lower.

Implant failure is a time-dependent problem and can be due to infection, microfractures, polyethylene wear, and soft tissue imbalance. Methods of evaluating the long-term results of prostheses and implant failure include functional scores [5],[6],[23] and aseptic loosening of implants [24]-[27]. A general consensus is that when daily activities cannot be performed as a result of problems due to the implant, a revision TKA is indicated. The results of this study indicated there was no difference in prosthesis survival in patients who underwent a staged or simultaneous bilateral TKA. This result is similar to that reported by Ritter et al. [6] who found that prosthesis failure was not different between patient who received simultaneous bilateral, stage bilateral, and unilateral TKAs.

The evaluation of complications is the largest limitation of data-based research, and in this study we were not able to evaluate the surgical and postoperative complications between the two groups. However, the unplanned readmission rates, which may serve as an indicator of serious complications, and the in-hospital mortality rates were similar between the two groups. Per NHIRD guidelines, a second hospitalization within 14 days after surgery may be directly associated with complications of the index surgery. Other study has shown that a second hospitalization within 90 days of the index surgery may indirectly be associated with complications of the surgery [18],[19]. Whether or not simultaneous bilateral TKA is associated with a greater incidence of complications than staged bilateral TKA remains uncertain and is probably closely related to patient selection. Two recent meta-analyses [2],[4] have suggested that simultaneous bilateral TKA is associated with a greater incidence of complications than stage bilateral TKA. Interestingly, the two analyses differed with respect to the complications that are more prevalent. A population-based study by Meehan et al. [7] examined complications occurring after staged bilateral (*n* = 23,715) and simultaneous bilateral (*n* = 11,445) TKA and reported that simultaneous bilateral TKA was associated with a significantly higher risk of myocardial infarction (adjusted odd ratio (aOR) = 1.6) and pulmonary embolism (aOR = 1.4), similar odds of death (aOR = 1.3) and ischemic stroke (aOR = 1.0), and lower odds of major joint infections (aOR = 0.6) and major mechanical malfunction (aOR = 0.7) than staged bilateral TKA. On the other hand, a number of studies with smaller patient numbers have reported a similar incidence of complications and similar outcomes between staged and simultaneous bilateral TKAs [9],[10]. The total hospital and outpatient charges likely reflect patient comorbidities and complications [4],[12],[14]. In our study, though there is a strong probability that there was a higher incidence of complications in the simultaneous bilateral groups, the overall costs of both inpatient and outpatient care associated with simultaneous bilateral TKA were lower than that of staged bilateral TKA; and the hospital unplanned readmission rates, which may serve as an indicator of serious complications, and the in-hospital mortality rates were similar between the groups.

A major limitation of this study is that disease comorbidity and postoperative complications could not be evaluated. Certain data that could not be analyzed included the brand of implant used and surgical parameters such as operation time, disease severity, and private payment of medical charges not covered by the NHI. Only revision surgery was considered with respect to a return to the operating room; limitations of the database prevented determining if other surgeries were related to the TKA or not. Revision TKA was specifically chosen because this indicates severe technique failure for which there is no other treatment. It should be noted that the length of hospital stays reported are normal for our country but are much longer than typical of many Western countries; thus, the results may not be generalizable to other countries where the length of stay is much shorter. Lastly, we did examine how quickly patients returned to their activities of daily life after surgery. Few studies have examined short-term functional recovery after the two procedures. Niki et al. [28] used a Knee Society function score of 80 as a target and reported that the mean recovery time to reaching this target was 2 months shorter with staged TKA than with simultaneous bilateral TKA. Our observations suggested that the patients who underwent a simultaneous bilateral operation recovered more slowly than those that received a staged procedure, but we did not use a metric to evaluate this question. This question certainly deserves future study.

## Conclusions

The results of this study indicate that simultaneous bilateral TKA is more cost effective than staged bilateral TKA. Prosthesis survival is not affected by the choice for staged or simultaneous bilateral TKA.

## Abbreviations

DVT: deep vein thrombosis

ICD-9-CM: International Classification of Disease, Ninth Revision, Clinical Modification

IQR: inter-quartile ranges

LHID: Longitudinal Health Insurance Database

LOS: length of stay

NHI: National Health Insurance

NHIRD: National Health Insurance Research Database

OA: osteoarthritis

RA: rheumatoid arthritis

TKA: total knee arthroplasty

USD: US Dollars

## Competing interest

The authors declare that they have no competing interests.

## Authors’ contributions

AC-CL is involved in literature research, clinical studies, experimental studies, statistical analysis, manuscript preparation, and manuscript editing. EC and H-CW are involved in data analysis and statistical analysis. C-MY is responsible for the study concepts, study design, and data acquisition. H-LM is responsible for the definition of intellectual content, literature research, and manuscript review. T-CL is the guarantor of the integrity of the entire study and is responsible also for the study design, data acquisition and manuscript review. All authors read and approved the final manuscript.

## References

[B1] McInnis DP Devane PA Horne G Bilateral total knee arthroplasty: indications and complications Curr Opin Orthop 2003 14 52 57

[B2] Hu J Liu Y Lv Z Li X Qin X Fan W Mortality and morbidity associated with simultaneous bilateral or staged bilateral total knee arthroplasty: a meta-analysis Arch Orthop Trauma Surg 2011 131 1291 1298 21359869 10.1007/s00402-011-1287-4

[B3] Raviraj A Chakravarthy M Pai S Prabhu A Comparison of simultaneous bilateral and staged bilateral total knee arthroplasty in terms of perioperative complications J Arthroplasty 2011 26 165 166 21093211 10.1016/j.arth.2010.08.005

[B4] Restrepo C Parvizi J Dietrich T Einhorn TA Safety of simultaneous bilateral total knee arthroplasty. A meta-analysis. J Bone Joint Surg Am 2007 89 1220 1226 17545424 10.2106/JBJS.F.01353

[B5] Ritter MA Harty LD Debate: simultaneous bilateral knee replacements: the outcomes justify its use Clin Orthop Relat Res 2004 428 84 86 15534524

[B6] Ritter MA Harty LD Davis KE Meding JB Berend M Simultaneous bilateral, staged bilateral, and unilateral total knee arthroplasty. A survival analysis J Bone Joint Surg Am 2003 85-A 1532 1537 12925634 10.2106/00004623-200308000-00015

[B7] Meehan JP Danielsen B Tancredi DJ Kim S Jamali AA White RH A population-based comparison of the incidence of adverse outcomes after simultaneous-bilateral and staged-bilateral total knee arthroplasty J Bone Joint Surg Am 2011 93 2203 2213 22159856 10.2106/JBJS.J.01350

[B8] Yoon HS Han CD Yang IH Comparison of simultaneous bilateral and staged bilateral total knee arthroplasty in terms of perioperative complications J Arthroplasty 2010 25 179 185 19195827 10.1016/j.arth.2008.11.103

[B9] Hutchinson JR Parish EN Cross MJ A comparison of bilateral uncemented total knee arthroplasty: simultaneous or staged? J Bone Joint Surg Br 2006 88 40 43 16365118 10.1302/0301-620X.88B1.16454

[B10] Stubbs G Pryke SE Tewari S Rogers J Crowe B Bridgfoot L Smith N Safety and cost benefits of bilateral total knee replacement in an acute hospital ANZ J Surg 2005 75 739 746 16173984 10.1111/j.1445-2197.2005.03516.x

[B11] Luscombe JC Theivendran K Abudu A Carter SR The relative safety of one-stage bilateral total knee arthroplasty Int Orthop 2009 33 101 104 17874240 10.1007/s00264-007-0447-1 PMC2899240

[B12] Reuben JD Meyers SJ Cox DD Elliott M Watson M Shim SD Cost comparison between bilateral simultaneous, staged, and unilateral total joint arthroplasty J Arthroplasty 1998 13 172 179 9526210 10.1016/s0883-5403(98)90095-x

[B13] March LM Cross M Tribe KL Lapsley HM Courtenay BG Cross MJ Brooks PM Cass C Coolican M Neil M Pinczewski L Quain S Robertson F Ruff S Walter W Zicat B Arthritis COST Study Project Group: Two knees or not two knees? Patient costs and outcomes following bilateral and unilateral total knee joint replacement surgery for OA Osteoarthritis Cartilage 2004 12 400 408 15094139 10.1016/j.joca.2004.02.002

[B14] Tien WC Kao HY Tu YK Chiu HC Lee KT Shi HY A population-based study of prevalence and hospital charges in total hip and knee replacement Int Orthop 2009 33 949 954 18612638 10.1007/s00264-008-0612-1 PMC2898996

[B15] http://nhird.nhri.org.tw/en/index.htm **National Health Insurance Research Database.** Available at . Accessed 4 Mar 2013.

[B16] Lin HC Xirasagar S Tang CH Costs per discharge and hospital ownership under prospective payment and cost-based reimbursement systems in Taiwan Health Policy Plan 2005 19 166 176 15070865 10.1093/heapol/czh020

[B17] http://www.health.gov.bc.ca/msp/infoprac/physbilling/payschedule/pdf/4-diagnostic.pdf Medical Services Commission: **July 2013 diagnostic and selected therapeutic procedures.** Available at . Accessed 14 Feb 2014.

[B18] Schairer WW Sing DC Vail TP Bozic KJ Causes and frequency of unplanned hospital readmission after total hip arthroplasty Clin Orthop Relat Res 2014 472 464 470 23801061 10.1007/s11999-013-3121-5 PMC3890213

[B19] Zmistowski B Hozack WJ Parvizi J Readmission rates after total hip arthroplasty JAMA 2011 306 825 826 21862739 10.1001/jama.2011.1182

[B20] Lai YS Wei HW Cheng CK Incidence of hip replacement among national health insurance enrollees in Taiwan J Orthop Surg Res 2008 1 42 18793382 10.1186/1749-799X-3-42 PMC2553065

[B21] Yang NP Deng CY Chou YJ Chen PQ Lin CH Chou P Chang HJ Estimated prevalence of osteoporosis from a Nationwide Health Insurance database in Taiwan Health Policy 2006 1 329 337 15946761 10.1016/j.healthpol.2005.04.009

[B22] Kovacik MW Singri P Khanna S Gradisar IA Medical and financial aspects of same-day bilateral total knee arthroplasties Biomed Sci Instrum 1997 33 429 434 9731398

[B23] Dennis DA Debate: bilateral simultaneous total knee arthroplasty Clin Orthop Relat Res 2004 428 82 83 15534523 10.1097/01.blo.0000147650.90507.84

[B24] Berend ME Ritter MA Hyldahl HC Meding JB Redelman R Implant migration and failure in total knee arthroplasty is related to body mass index and tibial component size J Arthroplasty 2008 23 6 Suppl 1 104 109 18722310 10.1016/j.arth.2008.05.020

[B25] Berend ME Ritter MA Keating EM Faris PM Crites BM The failure of all-polyethylene patellar components in total knee replacement Clin Orthop Relat Res 2001 388 105 111 11451108 10.1097/00003086-200107000-00016

[B26] Berend ME Harty LD Ritter MA Stonehouse DM 2nd Excisional arthroplasty for patellar loosening in total knee arthroplasty J Arthroplasty 2003 18 668 671 12934225 10.1016/s0883-5403(03)00202-x

[B27] Berend ME Small SR Ritter MA Buckley CA Merk JC Dierking WK Effects of femoral component size on proximal tibial strain with anatomic graduated components total knee arthroplasty J Arthroplasty 2010 25 58 63 19097851 10.1016/j.arth.2008.11.003

[B28] Niki Y Katsuyama E Takeda Y Enomoto H Toyama Y Suda Y Comparison of postoperative morbidity between simultaneous bilateral and staged bilateral total knee arthroplasties: serological perspective and clinical consequences J Arthroplasty 2014 29 504 509 23988436 10.1016/j.arth.2013.07.019

